# BMC Emergency Medicine reviewer acknowledgement, 2013

**DOI:** 10.1186/1471-227X-14-4

**Published:** 2014-02-13

**Authors:** Catia Cornacchia

**Affiliations:** 1 BioMed Central, Floor 6, 236 Gray's Inn Road, London, WC1X 8HB, United Kingdom

## Abstract

**Contributing reviewers:**

The editors of BMC Emergency Medicine would like to thank all our reviewers who have contributed to the journal in Volume 13 (2013).

## 

Abhishek Deshmukh

USA

Adi Lahat

Israel

A-Hyun Cho

South Korea

Albert Jacob Six

Netherlands

Alexandre Lautrette

France

Allan De Caen

Canada

Andreas Bur

Austria

Andrew Argent

South Africa

Anne-Maree Kelly

Australia

Asad Patanwala

USA

Bara Ricou

Switzerland

Barbara Latenser

USA

Bertrand Renaud

France

Brian Rowe

Canada

Brian Rayner

South Africa

Bronwyn Myers

South Africa

Bruno Pereira

Brazil

Bryan Bledsoe

USA

Carl Leier

USA

Christer Sandahl

Sweden

Chuck Heise

USA

Daniel Chan

Australia

David Lumenta

Austria

David Werring

UK

Deryk Ronald Chen

Trinidad and Tobago

Dimitrios Siassakos

UK

Douwe Postmus

Netherlands

Ebbe Billmann Thorgersen

Norway

Eric Lavonas

USA

Erica Frank

Canada

Esther Van Lieshout

Netherlands

Fabian Jaimes

USA

Gaye Moore

Australia

Georges Mion

France

Glenn Arendts

Australia

Hakan Yaman

Turkey

Hamid Shokoohi

USA

Hans Thulesius

Sweden

Homayoun Sadeghi-Bazargani

Iran

Ioana Bratu

Canada

Jaimie Meyer

USA

James Tsung

USA

James Bernat

USA

Jan G Zijlstra

Netherlands

Javad Salimi

Iran

Ji Hoe Heo

South Korea

Jim Lemon

Australia

John Odell

USA

Jonathan Samuel

Malawi

Joseph Carcillo

USA

Judith Hahn

USA

Karen Francis

Australia

Karim Brohi

UK

Katja Taxis

Netherlands

Katja E. Wartenberg

Germany

Khosro Hekmat

Germany

Koenraad Monsieurs

Belgium

Laurent Brochard

Switzerland

Leana Wen

USA

Marc Sabbe

Belgium

Margot Kushel

USA

Mark Mikkelsen

USA

Massimo Girardis

Italy

Mauro Mendlowicz

Brazil

Mehmet Topcuoglu

USA

Melanie Taylor

Australia

Melissa Bentley

USA

Michael David

USA

Michael Jones

UK

Nadia Abdala

USA

Nadine Schuurman

Canada

Nahathai Wongpakaran

Thailand

Nele Brusselaers

Sweden

Neville Shine

Australia

Olaf Aasland

Norway

Patrick Bodenmann

Switzerland

Peter Shirley

UK

Peter Aitken

Australia

Petr Patka

Netherlands

Philip Kolo

Nigeria

Pinchas Halpern

Israel

Pradeep Navsaria

South Africa

Raimondo Pavarin

Italy

Robert Hoesch

USA

Robert Booy

Australia

Robert Maunder

Canada

Sandra Leggat

Australia

Sandra Schneider

USA

Sang-Cheon Choi

South Korea

Sidgi Hasson

Oman

Steven Karch

USA

Tae Nyoung Chung

South Korea

Thomas Locker

UK

Thomas Kerr

Canada

Tolulope Oyetunji

USA

Toshiki Yokoyama

Japan

Ulrich Guller

Canada

William Pearson

USA

## Pre-publication history

The pre-publication history for this paper can be accessed here:

http://www.biomedcentral.com/1471-227X/14/4/prepub

